# Juvenile dermatomyositis is a different disease in children up to three years of age at onset than in children above three years at onset

**DOI:** 10.1186/1546-0096-10-S1-A63

**Published:** 2012-07-13

**Authors:** Anjali Patwardhan, Gloria C Higgins, Charles H Spencer, Robert M Rennebohm

**Affiliations:** 1 The Ohio State University and Nationwide Children's Hospital, Columbus, OH, USA; 2 University of Calgary and Alberta Children's Hospital, Calgary, AB, Canada

## Purpose

We tested the hypothesis that juvenile dermatomyositis (JDM) disease course in children with disease onset at or below age three years may be different than that of children with disease onset at greater than three years of age.

## Methods

Institutional Review Board approval was obtained to retrospectively review the charts of 78 patients with JDM seen in pediatric rheumatology clinic at Nationwide Children’s Hospital over the past 23 years. These patients were age 0-18 years with 19 patients at or below age three years at onset, and 59 above age three years at onset. The data regarding disease course and outcome were collected as of the last clinic follow-up or July30, 2010, whichever came first. Wilcoxon 2-sample test was used to compare continuous variables between the two age groups. Chi-square test and Fisher’s exact test were used to compare categorical variables between the two age groups.

## Results

The mean ages of onset in the two groups were 27 months and 91 months. The mean times between onset of symptoms to diagnosis in younger and older age groups were similar at 5.6 months and 4.5 months, respectively. The younger group had more females (p=0.05), were more likely to have a family history of autoimmune diseases (p=0.012), and were less likely to have disease onset during the typical winter-spring seasons (p = 0.031). The younger group was more likely to have a preceding fever (p=0.029), and less likely to have the following at diagnosis: heliotrope rash (p=0.04), Gottron’s sign (p=0.049), any rash (p=0.0495), nailfold capillary loop abnormalities (p=0.010), elevated creatinine kinase (p=0.022), elevated aspartate aminotransferase (p=0.021) and elevated aldolase (p=0.0353). Among those who had muscle biopsy at diagnosis, the younger children were more likely to have atypical histopathology (p=0.002). The younger group was treated more often with pulse methylprednisolone (p=0.0434), and less often with hydroxychloroquine (p=0.0351). There were no differences between the two groups in the initial oral corticosteroid dose (p=0.8017), treatment with methotrexate (p=0.709), and treatment with other immunosuppressants (p=0.323). There was no difference in the mean duration of methotrexate therapy (p=0.102), but the younger group had a shorter mean (p=0.038) and maximum (p=0.026) time on oral steroids. The two groups had similar proportions of patients with remission and active disease at 1 and 5 years. The younger patients were less likely to have active disease 10 years post diagnosis (p=0.019) and more likely to experience a monocyclic course (p=0.027).

## Conclusion

There were significant differences between JDM patients with disease onset at or below age three years, compared to their older counterparts. Younger patients in our cohort had fewer typical findings at diagnosis. They were more likely to experience a monocyclic course, a shorter total disease course, and a shorter duration of oral corticosteroid therapy.

## Disclosure

Anjali Patwardhan: None; Gloria C. Higgins: None; Charles H. Spencer: None; Robert M. Rennebohm: None.

